# Efficacy of chemotherapy for malignant pleural mesothelioma according to histology in a real-world cohort

**DOI:** 10.1038/s41598-021-00831-4

**Published:** 2021-11-01

**Authors:** Susana Cedres, Juan-David Assaf, Patricia Iranzo, Ana Callejo, Nuria Pardo, Alejandro Navarro, Alex Martinez-Marti, David Marmolejo, Alejandra Rezqallah, Caterina Carbonell, Joan Frigola, Ramon Amat, Anna Pedrola, Rodrigo Dienstmann, Enriqueta Felip

**Affiliations:** 1 Oncology Department, Hospital Universitari Vall d’Hebron & Vall d’Hebron Institute of Oncology (VHIO), Barcelona, Spain; 2 Thoracic Cancers Translational Genomics Unit, Vall d’Hebron Institute of Oncology (VHIO), Barcelona, Spain; 3 Oncology Data Science (ODysSey Group), Vall d’Hebron Institute of Oncology, Barcelona, Spain

**Keywords:** Cancer therapy, Mesothelioma

## Abstract

CheckMate 743 trial demonstrated survival benefit of immunotherapy in first line in MPM with some differences in the efficacy of chemotherapy according to histology. The objective of this study is to characterize the impact of chemotherapy according to histology in patients diagnosed with MPM at our institution. Clinical records of all MPM patients diagnosed at Vall d’Hebron University Hospital between November 2002 and April 2020 were reviewed. Associations between clinical variables and outcomes were assessed with Cox regression models. Survival data were calculated by the Kaplan–Meier method. 189 patients were included with 76% of tumors classified as epithelioid subtype. First line chemotherapy was offered to 85% of patients. Median survival in overall population was 21.3 months (95% CI 17.2–24.3). We found that patients with epithelioid tumors had better overall survival (OS) and progression free survival (PFS). Median OS of epithelioid patients treated with first line chemotherapy was 26.7 months versus 15.0 months in non-epithelioid patients (HR 2.25 CI 95% 1.4–3.4; p < 0.001). Median PFS for patients with epithelioid tumors treated with chemotherapy was 4.8 months versus 3.6 months in non-epithelioid (HR 1.5 CI 95% 1.0–2.3; p = 0.03). The improvement of outcomes in patients with epithelioid histology was detected in patients treated with cisplatin or carboplatin. Histology was not a predictive factor for the platinum agent sensitivity (p of interaction PFS = 0.09, p of interaction OS = 0.65). In our series, patients with non-epithelioid tumors presented worse prognosis. Although epithelioid tumors exposed to cisplatin had higher PFS, histology was not a clear predictor of chemotherapy efficacy.

## Introduction

Malignant pleural mesothelioma (MPM) is a rare and aggressive cancer arising from the mesothelial cells lining the pleura. Asbestos exposure is the major risk factor for mesothelioma with a very prolonged latency period between exposure to asbestos and the development of mesothelioma (20–50 years)^1^. The incidence rates of mesothelioma in the United States are 0.9 for men and 0.3 for women and in Europe 1.7 for males and 0.4 for females per 100,000 habitants^2^. The annual incidence of mesothelioma is increasing in Great Britain and Australia and it is predicted to increase in countries with poor regulation of asbestos mining.

Malignant mesothelioma is often refractory to standard chemotherapy regimens and exhibit poor prognosis, with overall survival being on the order of 9–18 months after diagnosis^3,4^. ECOG performance status, stage and histology are the strongest prognostic factors among patients with mesothelioma. The World Health Organization (WHO) classification includes three main histological subtypes (epithelioid, sarcomatoid and biphasic) with a different prognosis. Epithelioid histology is associated with a more favorable prognosis.

Treatment options for MPM patients who are not eligible for surgery are very limited. Platinum-based chemotherapy combined with an antifolate lead to a median survival of about 12–16 months^3,4^. The addition of bevacizumab or Tumor-Treating fields (TTF) to chemotherapy increases survival (18.8 and 18.2 months respectively)^5,6^. Carboplatin in association with pemetrexed is an accepted alternative option for patients who may not tolerate cisplatin^7,8^.

There remains an unmet clinical need for new, effective therapies that can improve outcomes in the first line. In recent years a dramatic improvement in advanced cancers therapy has been achieved with immune checkpoint blockade. However, results of early studies with immunotherapy in mesothelioma are contradictory, and currently Japan has approved nivolumab in second line setting and US and EU the combination of nivolumab plus ipilimumab in first line setting. Initial studies using single drug checkpoint inhibitors in previously treated patients demonstrated some efficacy with a median progression free survival (PFS) between 2 and 6 months with nivolumab and about 5 months with pembrolizumab^9–11^. However, the results of randomized trial are controversial^12–14^. Pembrolizumab and tremelimumab failed to show improvement in PFS and overall survival (OS) in second-third line versus chemotherapy or placebo, but recently the CONFIRM trial demonstrated that nivolumab improved PFS and OS versus placebo in relapsed MPM^14^. The combination of immune checkpoint inhibition with ipilimumab and nivolumab in previously treated patients demonstrated similar results for the combination and monotherapy in the MAPS2 trial, and in first line the combination demonstrated being superior to chemotherapy in the pivotal CheckMate 743 in terms of survival (OS 18.1 months) leading to the approval of this combination by FDA and EMA^15,16^. In a preplanned subanalysis considering histology, improvements in efficacy of immunotherapy over chemotherapy were statistically significant among those with non-epithelioid histology but not for epithelioid histology. Authors suggested that the differences were due to reduced efficacy of chemotherapy in non-epithelioid MPM. However, the pivotal trial which led to the approval of cisplatin plus pemetrexed in MPM did not evaluate the efficacy of chemotherapy by histology^3^. Moreover, ASCO guidelines published in 2018 did not recommend different systemic treatment according to histology and EURACAN/IASLC classification published in 2020 concluded that there is no clear evidence that chemotherapy provides less proportional benefit to patients with biphasic or sarcomatoid disease^17,18^. After the publication of CheckMate 743 we have review the clinical trials that had led to approval or recommendation of therapies in MPM (pemetrexed, raltitrexed, bevacizumab and TTFields). We found that the studies of pemetrexed and raltitrexed did not report the results by histology and in the trial with bevacizumab no differences were detected for histology. Finally, in a posthoc analysis in the study of TTFields patients with epithelioid tumors presented longer PFS. Only the combination of carboplatin plus pemetrexed reported no responses in sarcomatoid mesothelioma in a phase 2 trial. The aim of our study is to evaluate the efficacy of chemotherapy by histological subgroups in a real-world series of MPM patients.

## Methods

### Study design

This retrospective cohort study used real-world data from the electronic medical records from the Vall d’Hebron Hospital Universitari to identify patients with MPM who had initiated systemic therapy under routine clinical practice between February 2002 to February 2020. The study selection period encompasses the dates when immunotherapy was been evaluated in MPM, but not approved. Patients were followed longitudinally until death or their last visit prior to data cutoff. Demographic information, asbestos exposure, stage at initial diagnosis, sites of metastases, cancer treatment, medical history, disease characteristics and data on tumor evaluation (including progression of the disease and response to treatment), were considered as appropriate. Tissue specimens were obtained from the primary mesothelioma at the time of diagnosis by surgery or core biopsy. Each sample was assessed histologically for tumor tissue by two pathologists and cases were classified as epithelioid, sarcomatoid and biphasic subtypes of malignant mesothelioma. This study was approved by local ethical committee (Ethical Committee at Vall d’Hebron Hospital Universitary). Informed consent form was waived due to the retrospective nature of the study and permission for data usage was obtained from the local ethic committee (Ethical Committee at Vall d’Hebron Hospital Universitary). All methods were performed in accordance with the relevant guidelines and regulations.

### Patients

The study cohort included patients with confirmed MPM who had received at least one line of therapy for their disease between February 2002 to February 2020, had clinical record available and were 18 years or older. To allow for sufficient follow-up for clinical outcomes, patients entered the cohort no later than 10 months prior to data cutoff (March 2021). One hundred eighty-nine consecutive cases of MPM were retrospectively collected. Clinicopathologic information gathered included complete history, age, sex, performance status (PS), asbestos exposure, tumor stage and histological subtype. Neutrophil-to-lymphocyte ratio was calculated as the ratio between neutrophils and lymphocyte in the blood analysis obtained at the time of diagnosis. The tumor stage was defined according to the International Union Against Cancer´s tumor-node metastasis 8^th^ classification and sub-classified histologically according to the WHO guidelines^18,19^. All cases were reviewed by the local pathologists with expertise in the diagnosis of MPM. We evaluated a single tumor biopsy for each patient. All tumor biopsies analyzed were obtained by surgery (147 patients) or core needle biopsy (42 patients) and local pathologists confirmed the adequation of the sample to provide a diagnosis of MPM, histological subclassification and perform the needle immunohistochemical staining.

### Study outcomes

The primary objective of this study was to describe the association of the histology with overall survival (OS) and progression-free survival (PFS) in MPM who received systemic chemotherapy. Secondary analyses included assessment of the outcomes in patients treated with immunotherapy and a study of prognostic factors in a real-world series of MPM. OS for each patient was defined as the time to death from diagnosis of malignancy. Progression of the disease was determined by physician assessment based on radiographic evidence. PFS was defined as the time until the earliest record of actual disease progression or death from any cause from initiation of line therapy. PFS was analyzed by therapy type.

### Statistical analysis

Data were censored at last follow up for patients without relapse or death. Median follow-up time was calculated with reverse Kaplan–Meier estimator^20^. Median follow-up time was calculated with reverse Kaplan–Meier estimator. OS was calculated from diagnosis of malignancy until death due to any cause or until the date of last follow-up visit for still alive patients. Survival analysis that compared efficacy of chemotherapy by histology was carried out using the Kaplan–Meier curves and the significance was verified by a log-rank test. All p values were determined by two-sided tests and p values < 0.05 were considered significant. Multivariable analysis was done using the Cox regression model including only the clinical variables that showed significance in univariable analysis. A model with interaction between histology and platinum agent was constructed to determine whether the predictive value of chemotherapy agent is dependent on histology. Data analysis was produced by the R statistical software version 4.0.

## Results

### Patient population

We studied 189 patients with MPM whose clinicopathologic characteristics are summarized in Table 1. The median age was 68 years (range 45–88). Patients were predominantly male (70%), smokers (50%), had previous asbestos exposure (75%) and stage III (45%). The median neutrophil–lymphocyte ratio (NLR) was 5.2 and 58% have NLR less than 5. The total cohort comprised 145 epithelioid tumors, 17 sarcomatoid, 14 biphasic, and 13 cases with histological type not specified (10 of them obtained by thoracoscopy and 3 by an image-guided biopsy).

**Table 1:** Patients characteristics.

Baseline patients characteristics		
Characteristic	Number	Percentage
**Median age**	68 years (45–88)	
**Gender**		
Males	132	70
Females	57	30
**Performance status**		
0	44	23
1	131	69
2	14	13
**Asbestos**		
Yes	141	74
No	47	26
**Histology**		
Epithelioid	145	76
Non-epithelioid	44	24
**Stage**		
II	39	21
III	84	45
IV	59	33
**NLR**		
< 5	109	58
≥ 5	62	33
**First line chemotherapy**		
Yes	161	85
No	28	15
**Type of chemotherapy**		
Cisplatin-pemetrexed	102	66
Carboplatin-pemetrexed	32	27

*NLR* Neutrophil to lymphocyte ratio.

Out of the entire group, none of the patients was considered for resective surgery and 161 patients (85%) were treated with chemotherapy. Regarding the type of systemic treatment, 134 patients (84%) received platinum plus pemetrexed in first line. Among them 102 patients received cisplatin plus pemetrexed and 32 patients received carboplatin plus pemetrexed. Additionally, 16 patients (10%) were included in clinical trials in first line. The median number of cycles of chemotherapy in first line was 5 for patients treated with cisplatin or carboplatin. The objective response rate (ORR) to chemotherapy was 38% in epithelioid and 22% in nonepithelioid tumors with similar efficacy of chemotherapy in epithelioid patients treated with cisplatin or carboplatin.

### Survival analysis

**Figure 1:**
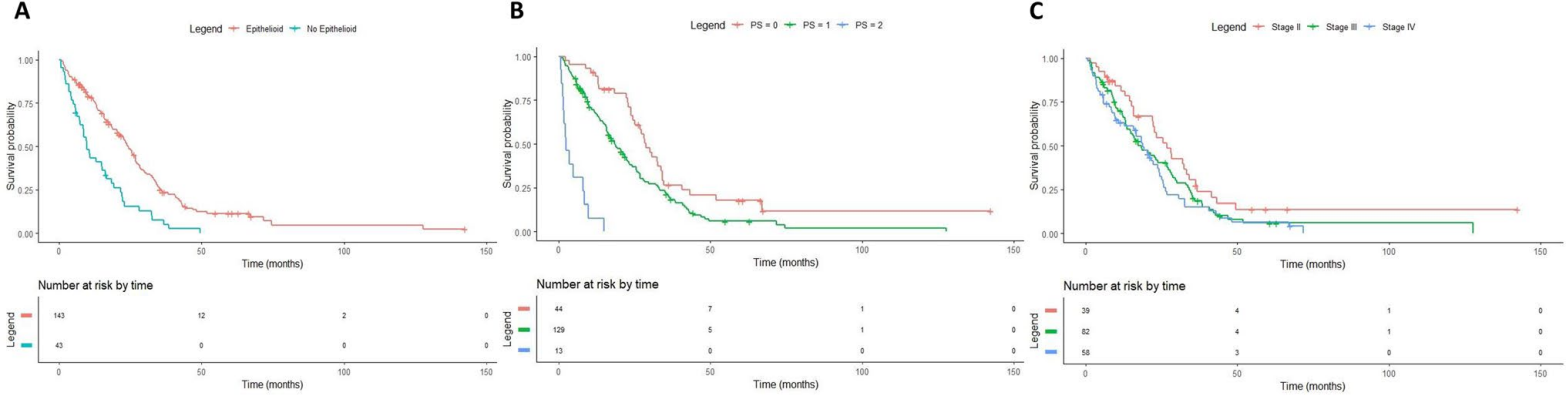
Kaplan–Meier overall survival according to histology (**A**), Performance Status (**B**) and clinical stage (**C**).

Median survival of the entire group was 21.3 months (95%CI 17.2–24.3 months). There was an improved survival rate in patients with good PS, epithelioid subtype histology, stage II and NLR < 5 (Fig. 1). Median survival for patients with PS0, 1 and 2 was 28.8 months, 18.8 months and 2.4 months respectively (p < 0.001). Median survival for patients with stage II was 28 months versus 18.4 months for patients with stage III/IV (p = 0.019 CI 95% 1.2–2.5). Patients with epithelioid histology had a median survival of 21.3 months versus 9.6 months in non-epithelioid patients (HR 2.4, CI 95% 1.6–3.4, p < 0.001). Median survival for patients with NLR < 5 was 25.1 months versus 12 months for patients with NLR > 5 (HR1.82, CI 95% 1.3–2.6, p < 0.001). We did not find differences in survival according to gender, smoking and asbestos exposure (p > 0.05). Median OS in patients which received first line systemic therapy was 21.6 months (19.1–25.2).

### Survival and type of treatment

**Figure 2:**
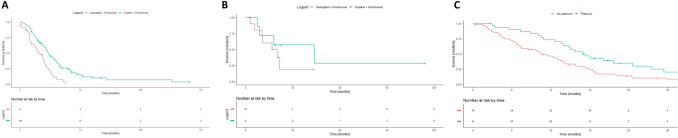
Kaplan–Meier overall survival according to type of systemic treatment: cisplatin versus carboplatin in first line (**A**), cisplatin versus carboplatin in second line (**B**) and platinum versus no platinum in second line (**C**).

We assess the magnitude of the treatment effect considering the type of treatment received. Median survival of patients treated in first line with cisplatin plus pemetrexed was 23.1 months versus 16.4 months for patients treated with carboplatin plus pemetrexed (HR 0.4, CI 95% 0.3–0.7, p < 0.001), Fig. 2. In second line, patients treated with cisplatin plus pemetrexed had a median OS of 43.7 months versus 18.5 months for patients treated with carboplatin plus pemetrexed (HR 0.5, CI 95% 0.1–1.9, p = 0.32). Median OS in second line was 17.1 months for patients who received platinum versus 10.7 months for patients treated without platinum agent (HR 0.5, CI 95% 0.3–0.8, p = 0.008).

In total 27 patients received cancer immunotherapy in second or further line (14 patients with anti-CTLA4, 8 patients with antiPD-1/PD-L1, 4 patients with anti-mesothelin and 1 patient with oncolytic virus). Median OS for patients treated with cancer immunotherapy was 26.4 months versus 20.9 months for patients that did not receive immunotherapy (HR 0.84, CI 95%0.5–1.3, p = 0.46).

### Survival and histology

**Figure 3:**
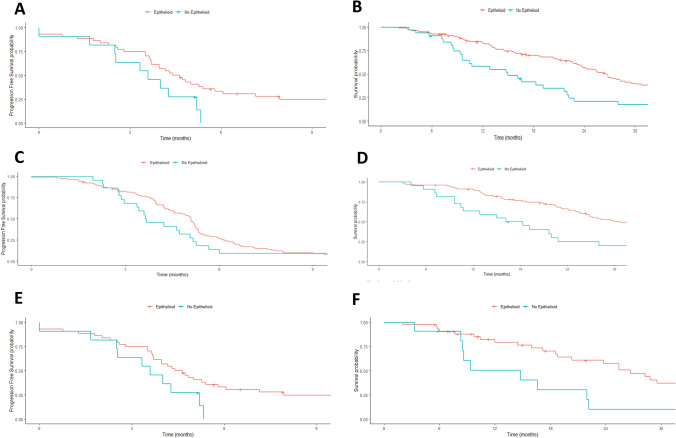
Kaplan–Meier overall survival according to histology: PFS and OS of patients treated with first line chemotherapy (**A**, **B**), PFS and OS of patients treated with cisplatin (**C**, **D**) and PFS and OS of patients treated with carboplatin (**E**, **F**).

In our series, patients with epithelioid subtype presented a median survival of 24.3 months versus 9.6 months biphasic, 8.6 months sarcomatoid and 20 months in no other specified histology (p < 0.001). When we analyzed the survival of patients who received first line chemotherapy according to histology, we found that patients with epithelioid tumors had better survival (Fig. 3). Median OS for patients with epithelioid tumors treated with chemotherapy in first line was 26.7 months versus 15.0 months in patients with non-epithelioid tumors (HR 2.25 CI 95% 1.4–3.4; p < 0.001). Analyzing all the histologies, median OS for patients treated with first line chemotherapy was 26.7 months in epithelioid, 11.2 months in biphasic, 10.7 months in sarcomatoid and 22 months in no other specified histology. Moreover, the PFS was also better for patients with epithelioid histology treated with first line chemotherapy (PFS 4.8 months versus 3.6 months in epithelioid and non-epithelioid patients respectively (HR 1.5 CI 95% 1.0–2.3; p = 0.03). Then we analyzed if the differences in survival according to histology were due to the type of systemic treatment received. The median OS for epithelioid patients receiving cisplatin plus pemetrexed was 30.7 months versus 17.2 months for non-epithelioid (HR 2.7 CI 95% 1.6–4.5; P < 0.001). For patients who received carboplatin plus pemetrexed in first line the median OS was 26.7 months in epithelioid versus 14.8 months in non-epithelioid patients (HR 2.7 CI 95% 1.3–5.8; p = 0.008). Median PFS was numerically higher in patients with epithelioid tumors who received cisplatin plus pemetrexed versus non-epithelioid population (5.1 months versus 3.6 months; HR 1.4 CI 95% 0.91–2.3; p = 0.11). Similarly, patients with epithelioid tumors treated with carboplatin had median PFS 4.5 months versus 3.6 moths in patients with non-epithelioid MPM (HR 1.99 CI 95% 0.96–4.1; p = 0.06). Despite the worse prognosis for non-epithelioid MPM, the interaction test with Cox regression model did not show significant value of histology as a predictive factor for the platinum agent sensitivity (OS, p interaction = 0.65, PFS p interaction = 0.09). (Table 2).

**Table 2:**
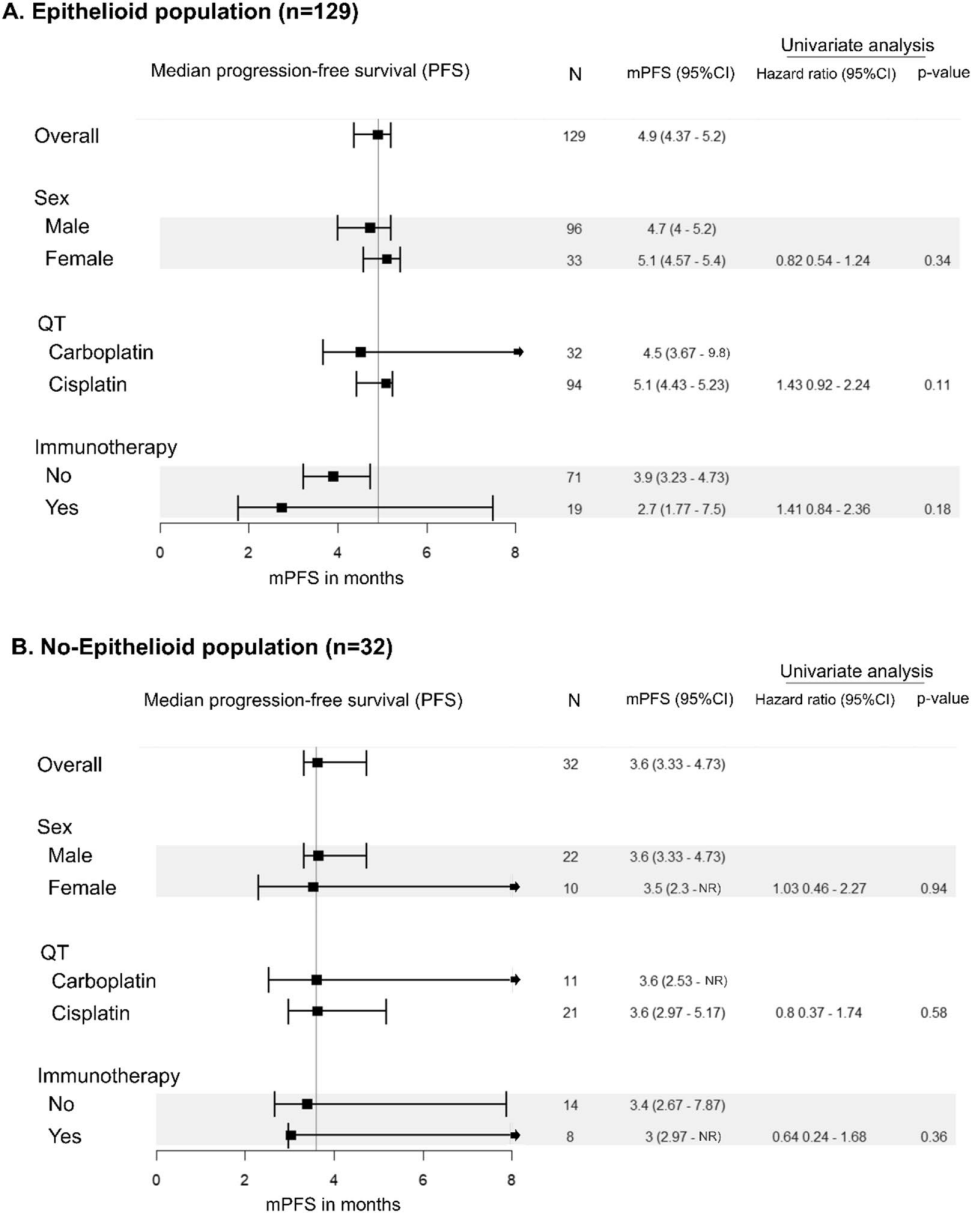
Multivariate analysis.

### Survival and cancer immunotherapy

**Figure 4:**
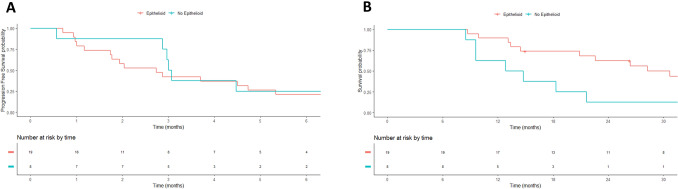
Kaplan–Meier Survival according to histology in patients treated with cancer immunotherapy: PFS (**A**) and OS (**B**).

Finally, we assess the impact of histology for patients treated with cancer immunotherapy. In total 27 patients were treated with cancer immunotherapy in second and further lines. Median OS for these patients treated with immunotherapy was 28.3 months for epithelioid versus 13.8 months for non-epithelioid patients (HR 3.4 CI 95% 1.3–8.7; p = 0.01). We did not detect difference in the PFS of patients treated with cancer immunotherapy in second line according to histology (2.7 months versus 3 months in non-epithelioid (HR 0.7 CI 95% 0.2–1.7; p = 0.43). (Fig. 4).

### Multivariable analysis

Using multivariable analysis with a Cox regression model that included significant variables in the univariate model, we found that epithelioid histology, NLR and treatment with cisplatin versus carboplatin remain significant prognostic factor for survival.

## Discussion

The aim of this study is to investigate the impact of histology in the efficacy of systemic treatment in a real-world database of MPM. Our results show that epithelioid tumors presented better outcomes when received chemotherapy, irrespective of the platinum agent.

Histology has been broadly studied in MPM and well recognized as a prognostic factor^21,22^. The two prognostic scoring systems classically used in mesothelioma were developed previous to the routine use of pemetrexed and both scores included non-epithelial histology as predictor of poor survival (EORTC and CALGB) with a difference in median survival around 3 months between epithelioid and non-epithelioid. However, the role of histology as predictive factor of efficacy to treatment with chemotherapy in advanced MPM has not been well address in the clinical trials. In the era of pemetrexed treatment, only a retrospective analysis of 131 patients treated with chemotherapy demonstrated that epithelioid histology was assessed with clinical benefit from first line chemotherapy^23^.

Recently, the study CheckMate 743 comparing chemotherapy versus nivolumab plus ipilimumab in untreated patients demonstrated that the combination of immunotherapy was superior in terms of survival^16^. In the trial patients were stratified by histology (epithelioid versus non-epithelioid) including 75% of patients with epithelioid histology. In a preplanned subanalysis, the improvement of immunotherapy over chemotherapy was clearly superior in non-epithelioid patients with a median OS of 18.1 months with immunotherapy and 8.8 months with chemotherapy. For the overall population no differences in terms of PFS were detected. Authors concluded that survival benefit with nivolumab plus ipilimumab over chemotherapy was observed regardless of histology with better outcomes for chemotherapy in epithelioid histology. The presence of PD-L1 also predicted improvement with nivolumab plus ipilimumab over chemotherapy, but according to the authors of the study, PD-L1 results were descriptive only, precluding definitive conclusions.

Since the publication of Checkmate 743, we purpose to evaluate if really exists difference in the efficacy of chemotherapy in MPM according to histology because in practice all patients are treated equally with the same chemotherapy without consider the histology as predictive factor. First of all, we have reviewed the information about subsets of histology and efficacy of chemotherapy published in clinical trials, but we could not find a clear difference in the outcomes (Table 3). The pivotal trial EMPHACIS published by Vogelzang demonstrated that combination of therapy with cisplatin plus pemetrexed was superior to cisplatin alone^3^. In the trial 68% of patients were epithelioid histology, but no data about the efficacy by histological subgroups were reported. Similarly, in a subsequent phase IV trial evaluating the role of pemetrexed in mesothelioma, the efficacy by histology was not reported^24^. Raltitrexed, the other antimetabolite approved in malignant mesothelioma also demonstrated improved in survival in combination with cisplatin and about two thirds of patients were epithelioid^25^. In this trial patients with non-epithelial tumors presented worse prognostic in univariate and multivariable analysis, but no data were published regarding the predictive role of the treatment with histology. Carboplatin in association with pemetrexed is an alternative option for patients who may not tolerate cisplatin^7,8^. In the larger of two randomized phase II studies response was observed in patients with epithelial or mixed histology, but no response was registered in patients with sarcomatoid mesothelioma^7^.

**Table 3:** Efficacy of treatment by histology in clinical trials.

	Pemet	Raltit	Carbop	Bevaciz	This series*	TTField	CM743 chemo	CM743 immuno	DREAM	PrE0505
**Epithelioid**										
OS	NR	NR	NR	NR	26.7	21.2	17	19	22	NR
PFS	NR	NR	NR	NR	4.8	8.3	NR	NR	7	NR
**Non-epithelioid**										
OS	NR	NR	NR	NR	15.0	12.1	9	18	7	NR
PFS	NR	NR	NR	NR	3.6	6.5	NR	NR	6	NR
**Global**										
OS	12.1	11.4	12.7	18.8	21.3	18.2	14.1	18.1	18.4	20.4
PFS	5.7	5.3	6.5	9.2	4.4	7.6	7.2	6.8	7.3	6.7

*Pemet* pemetrexed, *Raltit* raltitrexed, *Carbop* carboplatin, *Bevaciz* bevacizumab, *NR* not reported.

*OS of patients treated with 1st line chemotherapy, PFS in 1st line.

In order to improve the outcomes of the chemotherapy, the addition of antiangiogenics or TTFields has been explored. The MAPS trial demonstrated that survival was significantly extended with the addition of bevacizumab to chemotherapy^5^. In a preplanned subgroup analysis, the effect on survival of the bevacizumab containing regimen compared with standard chemotherapy was homogeneous when the analysis was stratified by histology subtype, moreover, the effect was more pronounced in patients with sarcomatoid or mixed histology (HR for OS of 0.82 (0.64–1.06) for epithelioid and 0.64 (0.40–1.02) for sarcomatoid). However, another two trials with antiangiogenic have failed in demonstrate benefit in patients with mesothelioma. The phase II LUME/Meso designed to assess the efficacy of nintedanib plus chemotherapy, demonstrated evident benefit in epithelioid histology, but not in biphasic, however the number of patients with biphasic histology was too low to provide a reliable estimate of the treatment effect^25^. Also, the addition of cediranib to chemotherapy improved PFS and there was no difference in the effect of treatment by histological subtypes^26^. In the STELLAR trial TTFields delivery system in combination with chemotherapy for first line leads to a median OS and PFS longer than historical control^6^. In a post-hoc analysis, OS and PFS were longer in patients with epithelioid histology than in patients with other subtypes (OS 21.2 vs 12.1 months and PFS 8.3 vs 6.5 months, respectively).

Less evidence of the difference in the efficacy of treatment according to histology has been evaluated in studies of previously treated patients. Vinorelbine has shown clinical activity in a phase II study and responses were observed in all three histologic subtypes of mesothelioma, including those with sarcomatoid and biphasic tumors^27^. More recently, lurbinectedin demonstrated no significant differences in PFS and OS concerning the impact of histology, suggesting that lurbinectedin is likely to equalize the prognosis of the mesothelioma subtypes^28^.

The impact of immunotherapy in mesothelioma has been recently demonstrated. Initial studies with monotherapy suggested efficacy, but randomized trials in previously treated patients are controversial. However, in first line setting, a recent study demonstrated better outcomes for immunotherapy over chemotherapy. In these studies of immunotherapy, predictive factors of response have been more studied. Keynote 028 phase I trial, enrolled previously treated PD-L1 positive mesothelioma patients and showed 40% of clinical benefit for more than 6 months^11^. In the trial 72% of patients were epithelioid but no results according to histology subtypes were reported. In the INITIATE trial, a single arm phase 2 trial of nivolumab plus ipilimumab, disease control rate at 12 weeks was achieved by 68%^9^. The study included 86% of patients with epithelioid subtype and the small number of tumors with non-epithelioid histology did not allow a meaningful comparison between histological subtypes. MAPS2 trial also evaluated the addition of ipilimumab to nivolumab and demonstrated better outcomes for the combination^15^. Patients were stratified by histology with 85% of patients being epithelioid and responses were reported in all histological groups. PROMISE-meso failed in demonstrate superiority of pembrolizumab over chemotherapy in relapsed mesothelioma^12^. In this trial patients with non-epithelioid tumors had a non-significant poorer PFS and OS for pembrolizumab as compared to epithelioid. Two studies testing the combination of chemotherapy plus durvalumab in first line have been reported. The Australian DREAM trial reported a 6 months PFS of 31%^29^. In the trial, 83% of patients were epithelioid and in a post-hoc analysis, responses were observed in all histological subtypes. The US PrE0505 trial also reported median OS of 20.4 months and this trial included 74.5% of patients with epithelioid tumors, but no data about the efficacy of the treatment by histology were reported^30^.

Since the publication of the CheckMate 743 pointing differences in efficacy of chemotherapy according to histology, we sought to perform a retrospective analysis of the efficacy of the chemotherapy with histology at our institution. We evaluated 189 patients and we found, in agreement with other series that histology is a strong prognostic factor with a difference in median OS of 11.7 months (21.3 months in epithelioid versus 9.6 months in non-epithelioid). In our real-world series, we could demonstrate that histology is a prognostic factor for PFS in favor of epithelioid histology in patients treated with chemotherapy with a median PFS of 4.8 versus 3.6 months (p = 0.03). We detected that patients with epithelioid histology treated with cisplatin had higher benefit than patients treated with carboplatin (4.5 months versus 3.6 months for patients treated with carboplatin). Despite the numerically higher PFS in patients with epithelioid tumors treated with cisplatin, there was no significant interaction between platinum agent and histology in Cox models, suggesting that histology is not a determinant of platinum agent sensitivity. Our results are in line with the clinical trials of chemotherapy and clinical guidelines, confirming that non-epithelioid patients had worse prognosis. However, the efficacy of chemotherapy in MPM is consistent in all histological subtypes (Table 3).

At the moment, the trials that led approval of pemetrexed and raltitrexed in malignant mesothelioma did not reported difference in efficacy of the chemotherapy according to histology, and the addition of bevacizumab demonstrated efficacy of treatment in all subgroups with better outcomes for sarcomatoid and biphasic tumors. Only the combination of carboplatin plus pemetrexed reported no responses in sarcomatoid mesothelioma. The more recent clinical guidelines available (ASCO, ESTRO/IASLC and NCCN) did not difference the type of chemotherapy considering histology. Our results, in accordance with previous studies confirms that histology is a prognostic factor. In our series epithelioid histology was a significant determinant of PFS in patients treated with chemotherapy, confirming one of the conclusions of the CheckMate 743 pointing worse efficacy of chemotherapy in non-epithelioid patients. In our study we included a small cohort of patients treated with immunotherapy (27 patients) and in this cohort we did not detect differences in PFS according to histology.

Our results have some limitations. This is a real-world series including all patients treated at one single institution. In our series, the number of epithelioid histology patients was high (76%) but this percentage is in line with the proportion of epithelioid patients included in clinical trials. We compare the impact of the treatment by histology but the number of patients with non-epithelioid included is small. Moreover, in our series we evaluated patients with tumor samples obtained by surgery and also by image-guided biopsies.

In conclusion, in our series we confirm that histology is a prognostic factor and patients with non-epithelioid tumors had worse survival. Patients with epithelioid histology presented better PFS than patients with non-epithelioid tumors. Ongoing studies combining checkpoint inhibitors plus chemotherapy are evaluating the impact of histology in the outcomes.
